# Multivendor comparison of global and regional 2D cardiovascular magnetic resonance feature tracking strains vs tissue tagging at 3T

**DOI:** 10.1186/s12968-021-00742-3

**Published:** 2021-05-13

**Authors:** Sebastian Militaru, Roman Panovsky, Vincent Hanet, Mihaela Silvia Amzulescu, Hélène Langet, Mary Mojica Pisciotti, Anne-Catherine Pouleur, Jean-Louis J. Vanoverschelde, Bernhard L. Gerber

**Affiliations:** 1 Division of Cardiology, Department of Cardiovascular Diseases, Cliniques Universitaires St. Luc UCL, Av Hippocrate 10/2806, 1200 Woluwe St. Lambert, Belgium; 2 Pôle de Recherche Cardiovasculaire (CARD), Institut de Recherche Expérimentale et Clinique (IREC), Université Catholique de Louvain, Brussels, Belgium; 3 International Clinical Research Center, St. Anne´S Faculty Hospital, Brno, Czech Republic; 4 1st Department of Internal Medicine/Cardioangiology, St. Anne´S Faculty Hospital, Faculty of Medicine, Masaryk University Brno, Brno, Czech Republic; 5 Philips Clinical Research Board, Paris, France

**Keywords:** Feature tracking, Tagging, Magnetic resonance imaging, Strain

## Abstract

**Background:**

Cardiovascular magnetic resonance (CMR) 2D feature tracking (FT) left ventricular (LV) myocardial strain has seen widespread use to characterize myocardial deformation. Yet, validation of CMR FT measurements remains scarce, particularly for regional strain. Therefore, we aimed to perform intervendor comparison of 3 different FT software against tagging.

**Methods:**

In 61 subjects (18 healthy subjects, 18 patients with chronic myocardial infarction, 15 with dilated cardiomyopathy, and 10 with LV hypertrophy due to hypertrophic cardiomyopathy or aortic stenosis) were prospectively compared global (G) and regional transmural peak-systolic Lagrangian longitudinal (LS), circumferential (CS) and radial strains (RS) by 3 FT software (cvi42, Segment, and Tomtec) among each other and with tagging at 3T. We also evaluated the ability of regional LS, CS, and RS by different FT software vs tagging to identify late gadolinium enhancement (LGE) in the 18 infarct patients.

**Results:**

GLS and GCS by all 3 software had an excellent agreement among each other (ICC = 0.94–0.98 for GLS and ICC = 0.96–0.98 for GCS respectively) and against tagging (ICC = 0.92–0.94 for GLS and ICC = 0.88–0.91 for GCS respectively), while GRS showed inconsistent agreement between vendors (ICC 0.10–0.81). For regional LS, the agreement was good (ICC = 0.68) between 2 vendors but less vs the 3^rd^ (ICC 0.50–0.59) and moderate to poor (ICC 0.44–0.47) between all three FT software and tagging. Also, for regional CS agreement between 2 software was higher (ICC = 0.80) than against the 3rd (ICC = 0.58–0.60), and both better agreed with tagging (ICC = 0.70–0.72) than the 3rd (ICC = 0.57). Regional RS had more variation in the agreement between methods ranging from good (ICC = 0.75) to poor (ICC = 0.05). Finally, the accuracy of scar detection by regional strains differed among the 3 FT software. While the accuracy of regional LS was similar, CS by one software was less accurate (AUC 0.68) than tagging (AUC 0.80, p < 0.006) and RS less accurate (AUC 0.578) than the other two (AUC 0.76 and 0.73, p < 0.02) to discriminate segments with LGE.

**Conclusions:**

We confirm good agreement of CMR FT and little intervendor difference for GLS and GCS evaluation, with variable agreement for GRS. For regional strain evaluation, intervendor difference was larger, especially for RS, and the diagnostic performance varied more substantially among different vendors for regional strain analysis.

**Supplementary Information:**

The online version contains supplementary material available at 10.1186/s12968-021-00742-3.

## Background

Myocardial strain imaging has become a widely popular tool for quantifying myocardial deformation, detecting subclinical disease, and obtaining prognostic information in various cardiac pathologies [1]. Cardiovascular magnetic resonance (CMR) tagging is considered the gold standard for strain assessment, but requires specific sequences and postprocessing software, and therefore has not been widely used outside of research studies. CMR feature tracking (FT) [2] is a novel post-processing approach that does not require additional image acquisition. This gives FT an advantage for use in a clinical setting, as it can retrospectively be applied to cine balanced steady state free precession (bSSFP) images, acquired using a clinically standard CMR protocol. Several software solutions are currently offering FT analysis using different motion tracking technologies [1], such as optical flow or non-rigid registration of cine bSSFP images. For speckle-tracking echocardiography (STE), significant dissimilarities between strain estimates performed by different ultrasound machine vendors and strain software packages have been observed in clinical studies [3–5], requiring efforts for standardization of deformation imaging between software packages to reduce intervendor variability [6]. Yet, so far, there have been only few intervendor comparisons of FT strains, all of them only performed using 1.5 T [7–11], and few validation studies evaluating its accuracy vs other techniques. Also, unlike STE or CMR tagging, FT cannot rely on physical markers of deformation in the myocardium, but only on endo and epicardial contour detection. Therefore, its accuracy to evaluate regional strains remains undefined.

Thus the aims of our study were: (1) to assess intervendor differences in CMR FT global, and particularly regional strain in cross-comparison versus tagging, the current gold standard for myocardial deformation measurement, and (2) to evaluate the accuracy of FT to measure regional strains and to detect myocardial akinesia in infarcted segments in patients with coronary artery disease (CAD). Hence, we compared 3 different CMR FT software vs CMR tagging and LGE in a population of 61 subjects with different cardiac pathologies.

## Methods

### Study population

The study protocol was previously published [12]. Subjects with various heart disease and healthy subjects were prospectively recruited after giving written informed consent to the IRB approved protocol (Comité Ethique Hospitalo Facultaire Université Catholique de Louvain, Brussels, Belgium). We screened two patient populations: (a) healthy subjects of both sexes and of different ages without any cardiovascular history, recruited by advertisement in the local community. Before inclusion and CMR, all self-reported healthy subjects underwent a clinical exam, assessment of medical history and cardiovascular disease risk factors, rest, and stress electrocardiogram (ECG), 2D echocardiography, and blood sampling. They were not eligible if they were pregnant or had any evidence of heart disease as indicated by clinical history, physical exam, or testing. (b) patients undergoing clinically indicated CMR for characterization of left ventricular (LV) hypertrophy (hypertrophic cardiomyopathy and aortic stenosis) or LV dysfunction (either ischemic heart disease or non-ischemic dilated cardiomyopathy). Exclusion criteria were atrial fibrillation or multiple premature beats and contraindication for CMR (pacemaker or other CMR incompatible implants, claustrophobia, severe renal failure). In the present study, we studied a subset of 61 randomly selected CMR studies of our total patient population. These were 18 healthy subjects (VOL), 18 patients with myocardial infarction (ISCH), 15 patients with dilated cardiomyopathy (DCM), and 10 patients with LV hypertrophy (LVH) either due to hypertrophic cardiomyopathy (n = 5) or aortic stenosis (n = 5).

### CMR acquisition

CMR studies were acquired using a 3 T CMR system (Achieva, Philips Healthcare, Best, Netherlands) as previously described [12]. We first acquired one set of conventional retrospectively electrocardiogram (ECG) gated bSSFP short-axis slices covering the LV and three 2- 3- and 4- long axis slices, respectively. Imaging parameters were: field-of-view 360 mm, slice thickness 8 mm, 2 mm spacing. flip angle 45 degrees, TR: 3.1 ms TE 1.5 ms, acquisition matrix 192 × 192 pixels, resulting in an acquired resolution of 1.9 × 1.9 mm reconstructed to 1.4 × 1.4 mm, SENSE factor 2, 25 acquired phases per cycle resulting in a temporal resolution of 25–40 ms. Then we repeated the acquisition of 8–10 short and 3 long-axis images using prospectively triggered cine hybrid gradient echo sequences with echoplanar read-out and grid spatial modulation of magnetization (SPAMM) Tagging in identical prescriptions to study myocardial deformation. Parameters were: field-of-view 36–40 cm; slice thickness 8 mm; spacing 2 mm; repetition time 7.2 ms; echo time 2.0 to 4.2 ms; flip angle 12°; echo-planar factor 7; matrix size 256 × 96–140; acquired temporal resolution 20 to 40 ms; tag spacing 7 mm. Then, 0.2 mmol/kg gadobutrol (Gadovist, Bayer Healthcare, Berlin, Germany) were injected and late gadolinium enhancement (LGE) images were obtained 10 min later in identical short and long axis prescription.

### CMR analysis

Images were anonymized on an Osirix workstation and analyzed by blinded observers. LV end-diastolic and end-systolic volumes, mass and ejection fraction (LVEF) were computed from the short-axis cine images and LGE was visually assessed and segments classified as non-infarcted or transmurally infarcted based on the presence and extent of LGE on post-contrast images using Segment (version 2.2, Medviso, Lund, Sweden http://segment.heiberg.se) as previously described [13]. Segmental LGE was classified visually by different degrees of transmurality (≥ 0%, ≥ 25%, ≥ 50%, ≥ 75% LGE) and will be referred to as “scar” in the following sections.

FT strain was computed with 3 different software (a) cvi42 (version 5.1, Circle Cardiovascular Imaging, Calgary, Canada), (b) Segment (version 3.0, Medviso) and (c) Tomtec Autostrain (Image Arena, version 4.6, Tomtec Imaging Systems, Unterschleissheim, Germany). All analyses were performed on the same image sets. For all software, initial user input is endocardial and epicardial contouring in one time frame, followed by automatic tracking and strain analysis, with the possibility of user correction of initial contouring and automatic reanalysis after visual assessment of correct tracking. There was no subjective difference in the amount of user corrections needed for different software. Peak systolic segmental longitudinal Lagrangian strain (LS) was computed on 4-, 2- and 3- chamber cine bSSFP images and circumferential strain (CS) and radial strains (RS) on the complete set of short-axis images. Segmental strain was recorded in a 16-segment model. We also computed global longitudinal strain (GLS), global circumferential strain (GCS), and global radial strain (GRS). Tagged images were analyzed using HARP software (Diagnosoft version 2.7, Diagnosoft, Inc., Baltimore, Maryland, USA) and segmental Lagrangian longitudinal, circumferential and radial peak systolic strains (denoted respectively as tagging GLS, tagging LS, tagging GCS, tagging CS, tagging GRS and tagging RS) were computed based on a 16-segment model, as cvi42 and Tomtec excluded the 17th apical segment in their analysis. The waveforms were filtered to remove large outliers and extended in end-diastole using linear extrapolation to compensate for the delayed acquisition of the first phase (about 30 ms after detection of the ECG R-wave peak time). GLS, GCS, and GRS were derived as a weighted average of the segmental waveforms, i.e., instead of each segmental waveform contributing equally to the global strain waveform, some segments contributed more than others to account for differences in segment lengths.

Global and regional midventricular Lagrangian longitudinal circumferential peak systolic strains (denoted respectively as GLS, LS, GCS CS, GRS and RS) were reported by convention with a negative sign for GLS/LS and CLS/CS representing myocardial shortening, and a positive sign for GRS/RS indicating myocardial stretching.

### Statistical analysis

The primary study endpoint was the comparison of peak midventricular FT against tagging on an intention to diagnose (including all segments irrespective of image quality).

Statistical analysis was performed using SPSS (version 21.0, Statistical Package for the Social Sciences, International Business Machines, Inc., Armonk, New York, USA) and R (version 3.3.2, R Foundation for Statistical Computing, Vienna, Austria). A p value < 0.05 was considered statistically significant. Data were tested for normality with Stem-Leaf plots, Histograms, and the Kolmogorov–Smirnov test. Continuous variables are presented as mean values ± SD, and categorical variables as counts and percentages. Comparisons of continuous and categorical baseline characteristics among groups of patients were carried out, respectively, using the Kruskal–Wallis test or the Ϫ^2^ test. Regional variation of strains in different segments in healthy volunteers was expressed as the coefficient of variation, and differences in CV among segments were compared using two-way repeated-measures ANOVA. Overall comparison between mean GLS GCS and GRS estimates by FT and tagging strains in healthy subjects and the whole group of patients was performed using the Kruskal–Wallis test. Individual comparisons between each software were performed using the Wilcoxon-U test with Bonferroni correction for multiple testing. Individual comparisons of regional and global strains between different FT software, and against tagging were performed using the two-way mixed-effects intraclass correlation coefficient (ICC) for overall agreement and Bland–Altman method for estimation of bias, as mean ± 2*SD. ROC curves were employed to evaluate the diagnostic capabilities of FT and tagging to distinguish different degrees of LGE scar transmurality (≥ 0%, ≥ 25%, ≥ 50%, ≥ 75%) vs non-infarcted segments in ISCH patients, and the area under ROC curves was compared using the nonparametric test according to the Delong method.

Intra- and inter-observer agreement for strain measurement was tested in 10 randomly selected cases according to the Bland–Altman method, and expressed as mean of absolute difference ± 2*SD, two-way mixed-effects ICC and coefficient of variation (CV).

## Results

### Clinical and CMR characteristics of patients

**Fig. 1:**
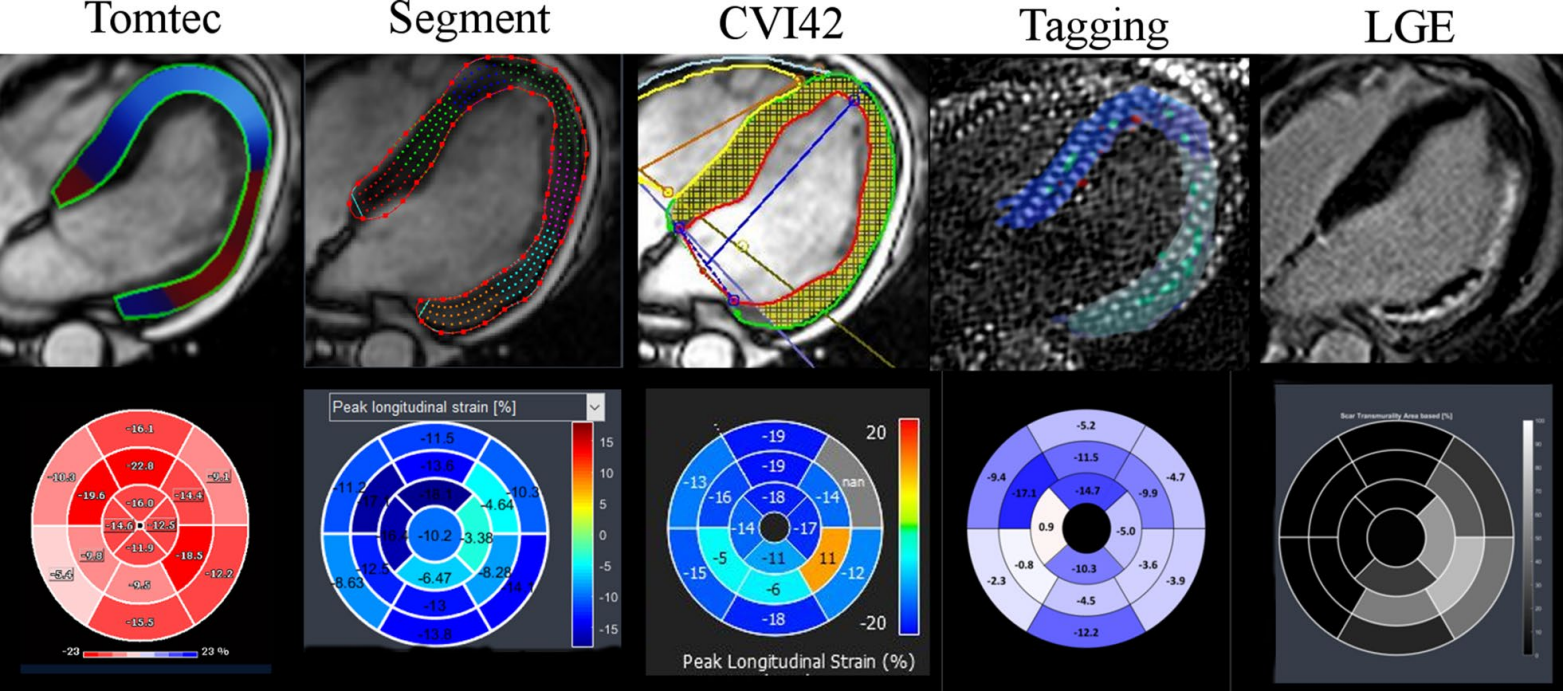
Example of strain analysis by tagging and the 3 feature tracking (FT) software in a patient with a lateral myocardial infarction

**Fig. 2:**
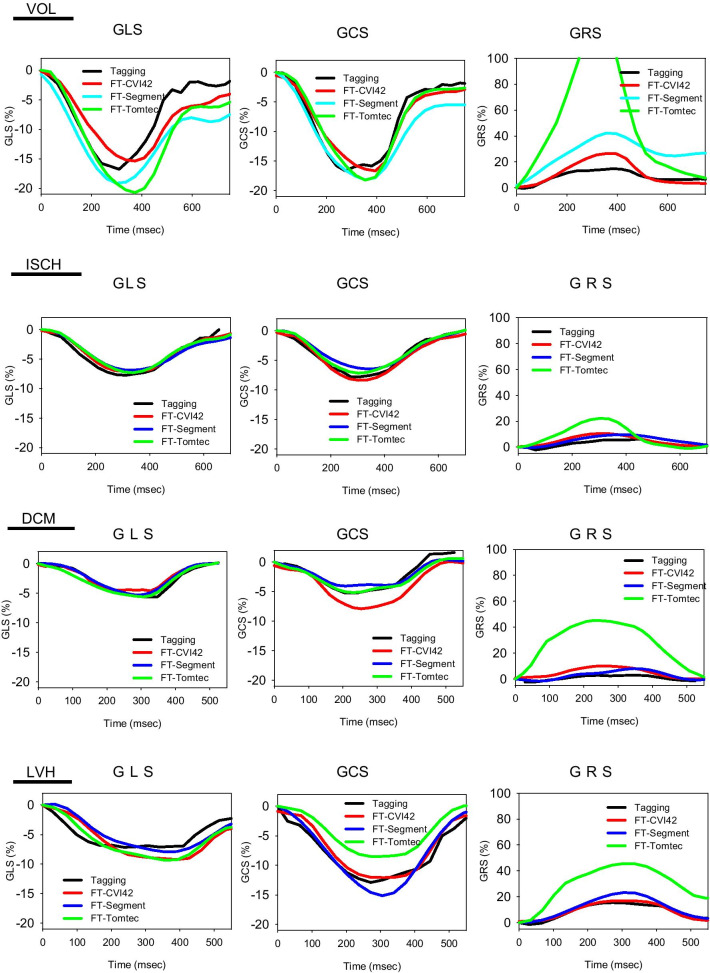
Example of global longitudinal strain (GLS), global circumferential strain (GCS) and global radial strain (GRS) in a typical healthy subject, a patient with myocardial infarction (ISCH), a patient with dilated cardiomyopathy (DCM) (c) and a patient with left ventricular cardiomyopathy (LVH)

The baseline characteristics of the study population are presented in Table 1. An example of strain maps by all four methods in a patient with a lateral infarction is shown in Fig. 1. Representative global strain curves in a healthy subject (VOL), a patient with infarct (ISCH), a patient with dilated cardiomyopathy (DCM), and a patient with LV hypertrophy (LVH) are shown in Fig. 2.

**Table 1:** Baseline and CMR characteristics of patients and volunteers

	All (N = 61)	VOL (n = 18)	ISCH (n = 18)	DCM (n = 15)	LVH (n = 10)	p value
Age (years)	53 ± 17	45 ± 16	60 ± 17	54 ± 18	55 ± 13	0.06
Male gender (n,%)	44 (72%)	10 (55%)	18 (100%)	9 (60%)	7 (70%)	0.015
Weight (kg)	73 ± 12	72 ± 10	76 ± 10	73 ± 18	69 ± 7	0.34
Height (cm)	172 ± 8	174 ± 9	174 ± 5	170 ± 9	168 ± 5	0.11
BSA (m2)	1.85 ± .17	1.86 ± .15	1.9 ± .14	1.84 ± 24	1.78 ± 0.9	0.17
Systolic BP (mmHg)	120 ± 19	123 ± 14	113 ± 23	119 ± 22	127 ± 15	0.27
Diastolic BP (mmHg)	74 ± 13	77 ± 9	67 ± 15	77 ± 16	77 ± 10	0.07
Heart Rate (bpm)	70 ± 13	66 ± 8	72 ± 11	70 ± 17	71 ± 18	0.52
LVEDV (ml)	229 ± 92	163 ± 32	290 ± 99	290 ± 59	145 ± 26	< 0.001
LVESV (ml)	144 ± 106	57 ± 14	217 ± 111	222 ± 56	49 ± 18	< 0.001
LVEF (%)	45 ± 23	65 ± 4	29 ± 17	24 ± 10	67 ± 8	< 0.001
LV mass (g)	132 ± 37	94 ± 18	150 ± 27	142 ± 31	140 ± 44	< 0.001

VOL, healthy subjects; ISCH, myocardial infarction subjects; DCM, dilated cardiomyopathy subjects; LVH, left ventricular hypertrophy subjects

BSA, Body surface area; BP, Blood pressure; LV, Left ventricle; EDV, End-diastolic Volume, ESV, End-systolic Volume, EF, ejection fraction

### Normal global and regional longitudinal and circumferential strain in healthy subjects

**Fig. 3:**
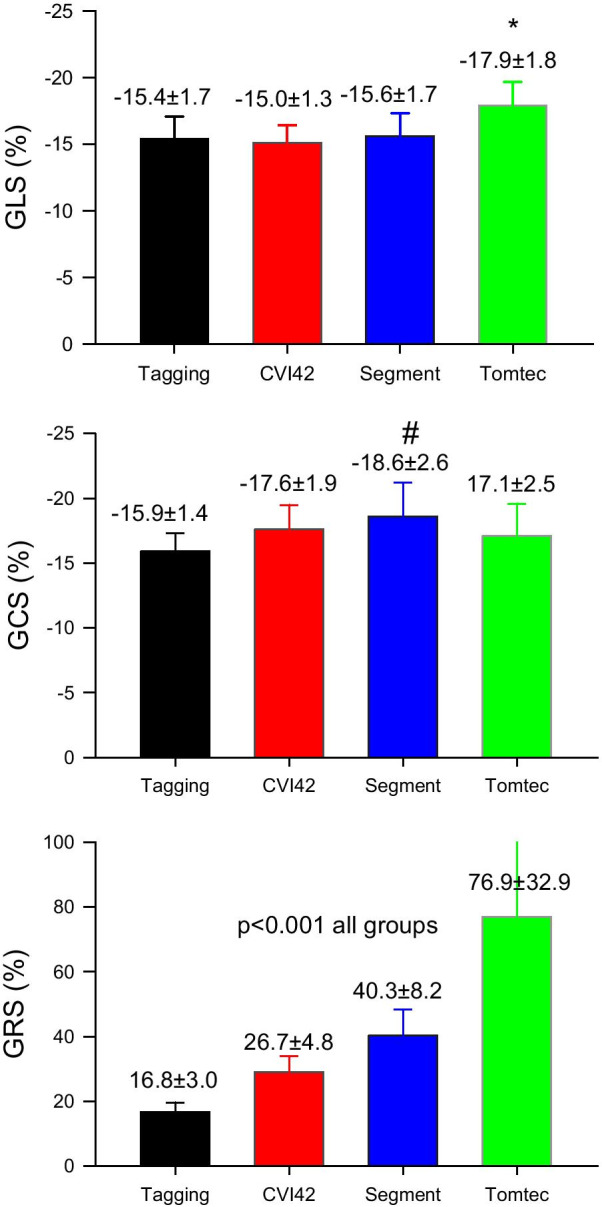
Average normal GLS GCS and GRS by different software in healthy subjects. *: p < 0.001 vs cvi42, Segment and tagging. #: p < 0.05 vs cvi42 and Tagging

Normal *global* strain values in the 18 healthy subjects and the other groups are shown in Fig. 3 and Table 2. In the healthy subjects GLS estimates by Tomtec software (− 17.9 ± 1.8%) were statistically greater (p < 0.001) than those by tagging (− 15.4 ± 1.8%, p <), cvi42 (− 15.0 ± 1.3%) and Segment (− 15.7 ± 1.7). On the other hand, GCS estimates were significantly (p < 0.05) higher by Segment (− 18.6 ± 2.6%) than by tagging (− 15.9 ± 1.4%), and cvi42 (− 17.6 ± 1.9%). However, the most important differences were those of GRS, whose estimates differed significantly among all vendors (+ 16.7 ± 3.0% by tagging, 29 ± 4.8% by cvi42, 40.3 ± 8.1% by Segment, 76.9 ± 32.9% by Tomtec, all individual comparisons p < 0.001).

**Table 2:** Global longitudinal and circumferential strain values by tagging and different feature tracking (FT) software

		All (n = 61)	VOL (n = 18)	ISCH (n = 18)	DCM (n = 15)	LVH (n = 10)	p value
GLS	Tagging (%)	− 9.7 ± 5.0^c^	− 15.4 ± 1.7 ^IDL c^	− 5.5 ± 2.8 ^Lc^	− 6.8 ± 3.3 ^L^	− 11.4 ± 3.6	< 0.001
	FT cvi42 (%)	− 10.1 ± 4.8^c^	− 15.0 ± 1.3 ^IDLc^	− 6.3 ± 3.3 ^L^	− 6.8 ± 3.5 ^L^	− 12.7 ± 2.6 ^c^	< 0.001
	FT Segment (%)	− 10.3 ± 5.3^c^	− 15.6 ± 1.7 ^ID^	− 6.5 ± 3.8 ^L^	− 6.6 ± 3.9 ^L^	− 13.2 ± 3.4	< 0.001
	FT Tomtec (%)	− 11.5 ± 5.9^c^	− 17.9 ± 1.8 ^ID^	− 6.9 ± 3.7 ^L^	− 7.2 ± 3.6 ^L^	− 14.7 ± 3.5	< 0.001
GCS	Tagging (%)	− 10.6 ± 4.5^ab^	− 15.9 ± 1.4 ^IDL b^	− 7.5 ± 2.4 ^L^	− 7.1 ± 3.2 ^L^	− 12.9 ± 1.6^ab^	< 0.001
	FT cvi42 (%)	− 12.0 ± 6.0	− 17.6 ± 1.9 ^ID^	− 7.3 ± 3.3 ^L^	− 7.0 ± 3.4 ^L^	− 17.2 ± 4.2	< 0.001
	FT Segment (%)	− 12.1 ± 6.8^c^	− 18.6 ± 2.6 ^ID^	− 6.8 ± 4.1 ^L^	− 6.7 ± 3.2 ^L^	− 18.5 ± 4.2^c^	< 0.001
	FT Tomtec (%)	− 11.0 ± 6.2	− 17.1 ± 2.5 ^ID^	− 6.5 ± 3.6 ^L^	− 6.0 ± 3.0 ^L^	− 15.6 ± 5.2	< 0.001
GRS	Tagging (%)^cab^	11.3 ± 5.6^cab^	16.7 ± 3.0 ^ID^	8.0 ± 2.5 ^Lbc^	6.0 ± 3.2 ^L^	16.2 ± 2.4^cba^	< 0.001
	FT cvi42 (%)^bc^	19.0 ± 11.9^bc^	29.2 ± 4.8 ^ID^	9.9 ± 5.1 ^Lc^	9.3 ± 5.2 ^L^	30.3 ± 11.0^bc^	< 0.001
	FT Segment (%)^c^	25.1 ± 16.7^c^	40.3 ± 8.1 ^ID^	12.5 ± 9.3 ^Lc^	12.6 ± 9.3 ^L^	38.6 ± 9.5^c^	< 0.001
	FT Tomtec (%)	53.6 ± 38.0	76.9 ± 32.9 ^ID^	30.0 ± 43.7 ^L^	45 ± 20	73.4 ± 24.9	< 0.001

GCS, global circumferential strain; GLS, global longitudinal strain; GRS, global radial strain

Paired comparison within each test I: p < 0.05 vs ISCH, D: p < 0.05 vs DCM, L: p < 0.05 vs LVH

Paired comparisons among tests: ^a^p < 0.05 vs cvi42 ^b^p < 0.05 vs Segment ^c^p < 0.05 vs Tomtec

**Fig. 4:**
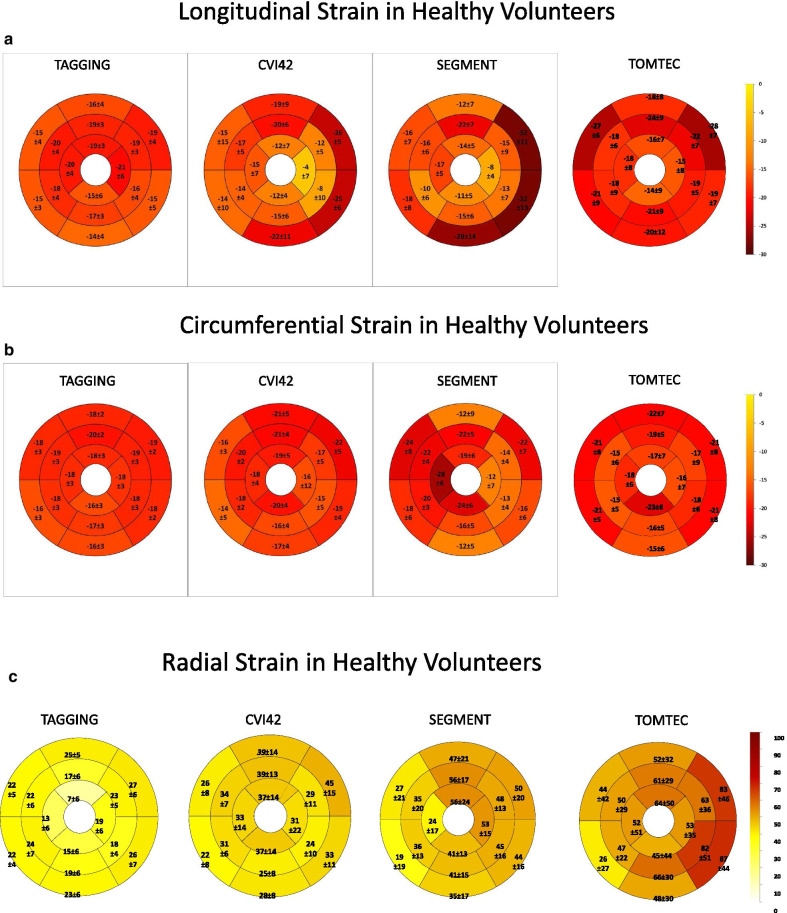
Bullseye showing the mean ± SD of normal values of regional longitudinal strain (LS) (**a**) circumferential strain (CS) (**b**) and radial strain (RS) (**c**) values by tagging and the 3 different FT software in healthy subjects

Bullseye plots showing average and SD of normal *regional* LS, CS, and RS in healthy subjects by the 3 FT software and tagging are depicted in Fig. 4a, b, and c. Coefficients of variation among segments in healthy subjects in Table 3 demonstrate that normal regional LS and CS were variable among LV planes by all modalities (p < 0.001 by ANOVA). For all modalities, this regional variability was lower for CS than for LS and RS. Also, for each strain direction tagging had less regional variability than FT methods.

**Table 3:** Variability (coefficient of variation) of regional strain among segments in healthy volunteers

	Tagging (%)	cvi42 (%)	Segment (%)	Tomtec (%)
Longitudinal strain	25	61	61	44
Circumferential strain	16	30	42	38
Radial strain	38	42	49	70

### Global longitudinal, circumferential, and radial strain

GLS, GCS and GRS values by tagging and FT in healthy subjects and different groups of patients are shown in Table 2. For all modalities, GLS, GCS and GRS were significantly lower in ISCH patients and DCM than in VOL for all modalities. However, the differences in GLS between VOL and LVH were only significant for tagging and cvi42 but not for Segment and Tomtec. Also, GCS estimates between VOL and LVH patients were only significantly different for tagging, but not for any of the other three FT software.

**Fig. 5:**
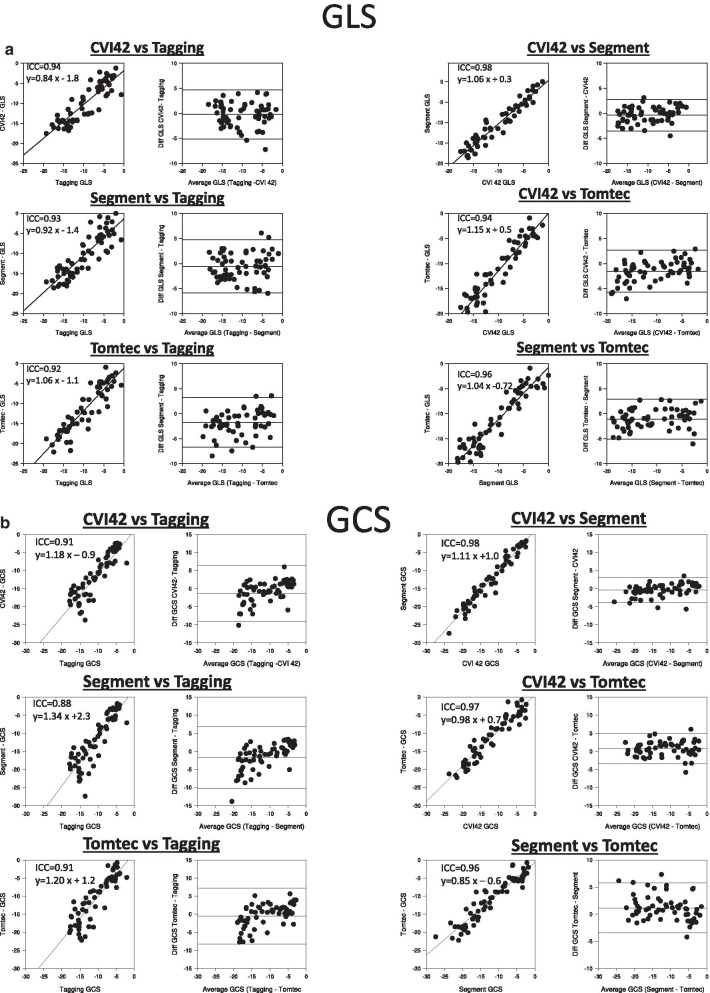

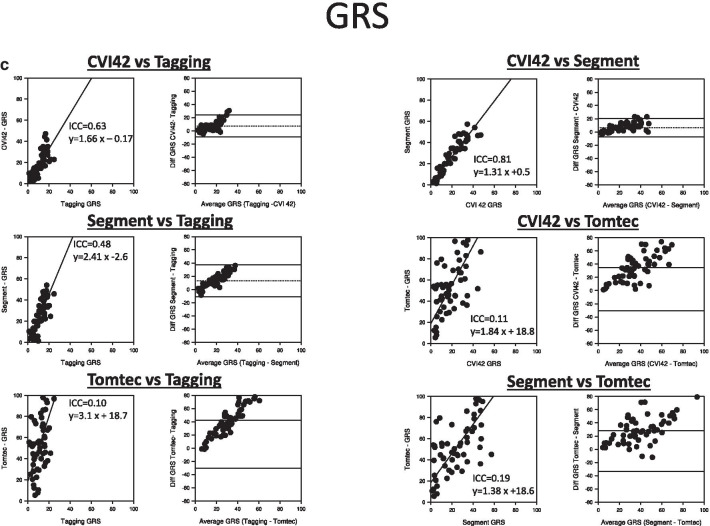
Scatter and Bland–Altman plots for comparisons between (**a**) GLS and (**b**) GCS by different FT software against tagging and among each other

Correlation and Bland Altman plots for GLS GCS and GRS among different FT software and against tagging are shown in Fig. 5a–c. The agreement among the three FT software was excellent for both GLS (ICC between 0.94 and 0.98) and GCS (ICC between 0.96 and 0.98). Also, both GLS (ICC between 0.92–0.94) and GCS (ICC between 0.88–0.91) estimates of each of the three FT software had an excellent agreement with tagging. There was no significant bias between GLS estimates by cvi42 and tagging (− 0.2 ± 2.4, 95% CI [− 0.8; 0.4]), between cvi42 and Segment (− 0.3 ± 1.6 95% CI [− 0.7;0]) nor between Segment and tagging (− 0.6 ± 2.7, 95% CI [− 1.2;0.1]). However GLS values by Tomtec were lower and this bias in GLS values by Tomtec vs tagging (− 1.8 ± 2.5, 95% CI [− 2.4;− 1.1], p < 0.001)), cvi42 (− 1.5 ± 2.1, 95% CI [− 2.1;1.0], p < 0.001) and Segment (− 1.2 ± 2.0, 95% CI [− 1.7;− 0.7, p < 0.001) was significant. For GCS, cvi42 and Segment provided higher values, and significant bias against the other software (− 1.3 ± 3.8, 95% CI [− 2.2; − 0.3], p < 0.005 CVI42 vs tagging, − 0.8 ± 2.1, 95% CI [− 0.2;− 1.3] cvi42vs Tomtec, p < 0.001, -1.7 ± 4.3, 95% CI [− 2.7; 0.6), p < 0.005 Segment vs tagging and − 1.1 ± 2.2, 95% CI [− 1.7; − 0.6], p < 0.001 Segment vs Tomtec, respectively). Moreover, this bias was skewed and increased for higher GCS values. GCS values of cvi42 and Segment (− 0.4 ± 1.8, 95% CI [− 0.8; 0.1], p = 0.16) or between Tomtec and tagging (− 0.5 ± 3.9, 95% CI [− 1.5; 0.5], p = 0.58) were however not significantly different. For GRS, the agreement between cvi42 and tagging (ICC = 0.63) and between Segment and tagging (ICC = 0.48) were acceptable. However, both aforementioned FT software had significant bias (7.4 ± 8.3, and 13.3 ± 12.1, respectively, both p < 0.001) vs tagging. Agreement between GRS by Segment and cvi42 (ICC = 0.81) was high, but also had significant bias (6.4 ± 6.8, p < 0.001) between methods. On the other hand, the agreement between all methods and Tomtec was poor (ICC 0.10–0.19), and Tomtec significantly provided significantly higher GRS than the 2 other FT vendors and tagging.

### Regional strain comparison

**Fig. 6:**
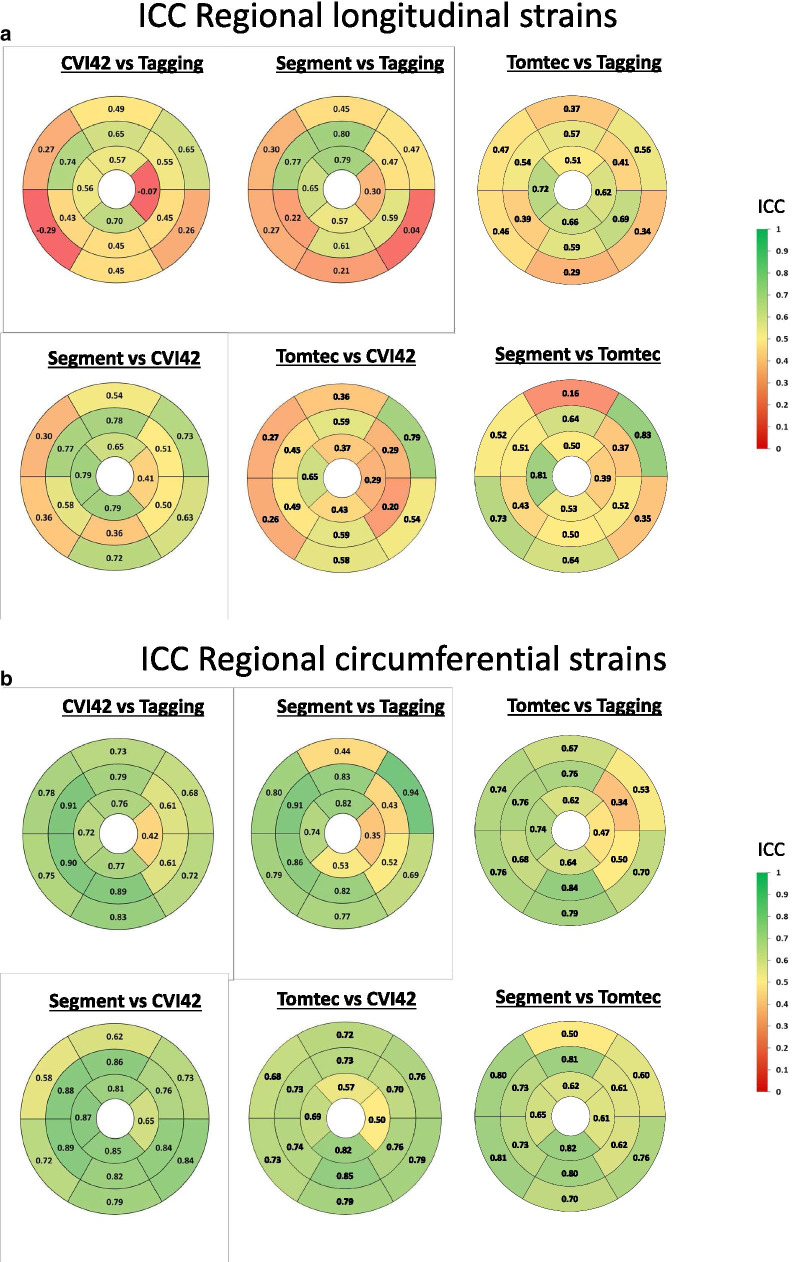

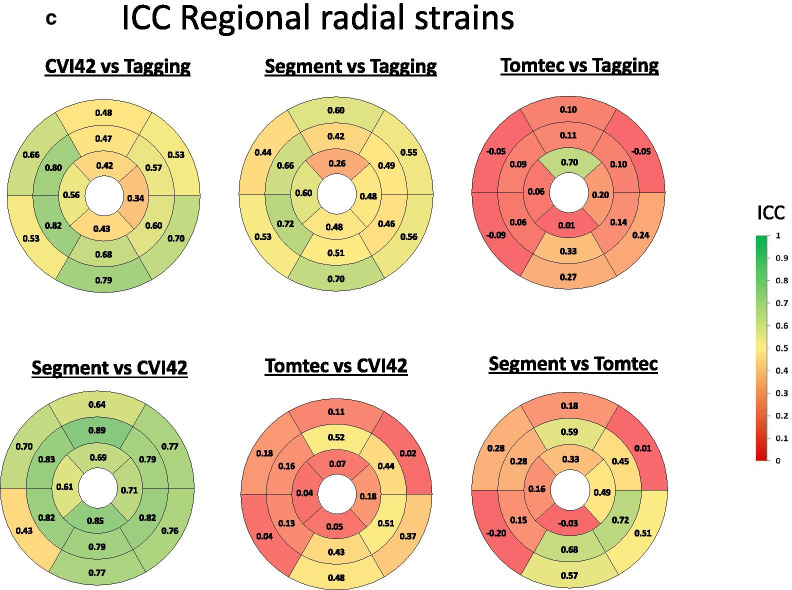
Bullseyes graphs showing the intraclass correlation coefficient at regional level between FT and tagging for LS (**a**), CS (**b**) and RS (**c**) in the study population

Agreement between regional LS, CS and RS is shown in Fig. 6a–c and bias is provided in Additional file 1: Figs. S1a–c. For LS, the overall agreement at the regional level was higher between cvi42 and Segment (ICC 0.68) than between Tomtec and cvi42 (ICC = 0.49) or Segment (ICC = 0.59), respectively. Also, the overall agreement was only moderate between all 3 FT software and tagging (cvi42 vs tagging ICC = 0.45, Segment vs tagging ICC = 0.44 Tomtec vs tagging ICC = 0.50). As shown in Fig. 6a, there were regional differences in the agreement of FT vs. tagging. Indeed, all 3 FT software agreed less well with tagging in basal segments than in apical segments. In contrast, among CMR FT software, the regional agreement was more variable and tended to be best in infero- and latero-basal segments and in the mid-anterior segment. The bias of different FT software vs tagging was higher in the lateral, infero-lateral and infero-basal segments, whereas it was worse among software in mid-lateral and anteroseptal basal segments (Additional file 1: Fig. S1a).

For regional CS, the overall agreement was high and again better between cvi42 and Segment (ICC 0.81) than between cvi42 and Tomtec ICC = 0.60) or Segment and Tomtec (ICC = 0.58). Also, overall agreement of the 3 FT software and tagging was high (ICC 0.77, 0.72 and 0.57 for cvi42, Segment and Tomtec, respectively). Figure 6b illustrates that regional agreement between all software was overall similarly high for all segments. Also, the bias for regional CS was homogeneously distributed among segments (Additional file 1: Fig. S1b).

Finally, for RS, the overall agreement was high between Segment and cvi42 (ICC = 0.75, p < 0.001), while it was poor between cvi42 and Tomtec (ICC = 0.16, p < 0.001) and between Segment and Tomtec (ICC = 0.23, p < 0.001). The agreement of regional RS for cvi42 and Segment vs tagging was acceptable (respectively ICC = 0.51 and ICC = 0.45, p < 0.001), but absent between Tomtec and tagging (ICC = 0.05, p = 0.19). The agreement between Segment and cvi42 was homogeneous among segments, whereas the agreement between cvi42 and Segment vs tagging was better for midventricular and basal inferior segments. The bias for regional RS between cvi42 and Segment vs tagging and between Segment and cvi42, respectively, was higher for anterior and lateral segments, (Additional file 1: Fig. S1c).

### Accuracy for scar detection

**Fig. 7:**
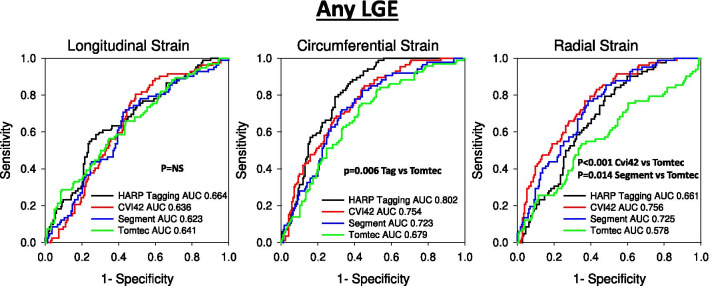
Receiver operating characteristics curve analysis comparing diagnostic abilities of detection of scar (any LGE) of infarcted segments by regional LS, CS, and RS by tagging and the 3 FT software

The accuracy of segmental strain to distinguish between the presence of any segmental scar (LGE of any transmurality) was assessed in the 18 patients with chronic infarct is shown in Fig. 7. Accuracy for other degrees of transmurality is shown in Additional file 2: Figs. S2a, b, and c.

For LS, all FT software had similar high AUC, and there was no significant difference in AUC between any of the FT software and tagging for any level of transmurality of LGE (> 0%, ≥ 25%, ≥ 50%, ≥ 75%). By contrast, for CS, tagging better discriminated infarcted vs. non-infarcted segments of any degree of LGE transmurality with higher AUC than Tomtec. Also, cvi42 better discriminated infarcted segments ≥ 25%, ≥ 50% and ≥ 75% than Tomtec. There were no statistically significant differences of AUC between CS by tagging and cvi42 for any level of transmurality of LGE. Segment had intermediate AUC that was not statistically different from tagging or Tomtec). Finally, for RS both cvi42 and Segment had the highest AUC to discriminate infarcted vs. non-infarcted segments of any degree of LGE transmurality and both were significantly higher than Tomtec. Tagging had significantly higher AUC than Tomtec only for detecting segments with ≥ 75% transmurality (Additional file 2 Fig S2c).

### Intra and interobserver reproducibility of strain measurements

The intra and inter-observer variability for global strain measurements is shown in Table 4. For all techniques and software, the inter and intraobserver reproducibility was excellent.

**Table 4:** Intra and interobserver reproducibility of global strain measures

		Intraobserver variability		Interobserver variability	
		ICC	CV (%)	ICC	CV (%)
Tagging	GLS	0.99	7	0.86	10
	GCS	0.95	12	0.93	10
	GRS	0.99	6	0.98	16
cvi42	GLS	0.94	14	0.90	15
	GCS	0.99	9	0.97	10
	GRS	0.95	20	0.95	15
Segment	GLS	0.99	5	0.95	11
	GCS	0.98	11	0.96	11
	GRS	0.96	20	0.80	36
Tomtec	GLS	0.96	12	0.97	13
	GCS	0.97	11	0.97	11
	GRS	0.91	34	0.76	27

ICC, Intraclass correlation coefficient; CV, coefficient of variation

## Discussion

Our study evaluated intervendor differences and comparison of CMR FT global and regional strains vs tagging at 3T.

We observed that GLS and GCS had an excellent agreement between each of the three FT software and tagging. However, there were small, albeit, significant differences in absolute values. Indeed, Tomtec provided slightly higher GLS values than the other 2 FT vendors. On the other hand, the GCS values by cvi42 and Tomtec were slightly greater than those by tagging and Segment. These differences in GLS and GCS were minor, and probably are negligible in clinical practice. By contrast, the intervendor difference for GRS was substantial. Whereas the agreement in GRS between cvi42 and Segment was good, it was poor against Tomtec. Also, there was a very important difference in GRS values among all FT software. Tomtec GRS values were approximately two times higher than those by Segment, which were again almost 25% higher than those by cvi42. Also, FT GRS was always significantly greater than tagging. The high agreement of CMR FT GLS and GCS strains among different vendors and against tagging corroborates other works comparing FT to other methods such as tagging [7, 8, 14–21], SENC [8, 15] or DENSE [7, 22]. Also, intervendor difference [7, 10] and mild overestimation of global FT strains vs tagging and in particular substantial differences in GRS [7, 10, 16, 19] have been previously reported [8, 19, 20, 22]. For GCS, we observed that overestimation increased at high values, similar to earlier comparisons of GCS by 2D [12] or 3DSTE [23] relative to tagging. A possible explanation is that tagging may underestimate high strains [24] due to lower temporal resolution than FT images, and due to the time lag for tag deposition at the beginning of systole. There are several potential explanations for the differences in radial strains. Because at a tag spacing of 6 mm only maximum 2 tag lines can cross the thickness of the LV wall, tagging could be less accurate for the estimation of RS than for other strain directions and might underestimate true radial thickening. Also, we assume that the different FT vendors compute RS differently. Tomtec, as opposed to the 2 other vendors, likely reports wall thickening from endo to epicardium, rather than averaged myocardial RS. Another explanation may be that the exact detection of endocardial and epicardial layers plays a more significant role in FT-RS estimates than for other strains.

Our study also evaluated *regional FT strain* differences. We found that regional peak-systolic LS CS and RS in healthy subjects were less homogenous for all 3 FT software than for tagging. Also, there was more variability in the agreement between regional strain measurements among FT software. The agreement for regional strains between cvi42 and Segment was higher than against Tomtec. Also, the agreement was better for regional CS and LS than for RS. Whereas all three FT software had an acceptable agreement with tagging for regional CS, the agreement of regional LS and CS by the three FT software with tagging was substantially worse. Although there have been only a few studies comparing regional strains by FT [8, 19, 25, 26], such modest agreement for segmental strains with other methods has also been reported before. However, there has so far only one study evaluating intervendor variability of regional strain by multiple FT software (Segment, cvi42, Tomtec, and Medis) [11], which exposed that cvi42 had the widest confidence interval for all three measurement types (longitudinal, circumferential and radial). This was not confirmed in our study. However, the reference they used was the mean regional strain of all vendors, whereas, in our study, tagging was used as a reference, and we compared each FT vendor on a one-on-one comparison. This difference in approach may explain the opposing findings: in our study cvi42 scored highest in both LS and regional CS analysis compared to tagging and had a high correlation with Segment (which was not assessed in the aforementioned study). Also, in contrast to our earlier observations with STE [23], we did not observe a significant increase of regional LS and particular CS strains from the base to the apex for FT. Finally, we also compared the accuracy of regional strains for the detection of LGE. We found that the accuracy for scar detection of regional LS was lower than that of regional CS and RS. For LS, there was no significant difference in accuracy among the 3 FT software or between any software and tagging. However, for CS and RS, we observed a significant difference in accuracy between vendors. CS by tagging had higher accuracy than Tomtec, whereas RS by cvi42 and Segment had higher diagnostic accuracy than Tomtec to identify scar. These findings are in line with those by Dobrovie et al. [11], who reported that regional CS by cvi42 and Medviso had the highest area under the curve for infarct detection [11]. However, in contrast to that study, where no intervendor difference in scar detection was reported for RS, in our study segmental RS by cvi42 and Segment had higher discrimination for detection of scar than Tomtec.

Differences in algorithms and other unknown constraints likely explain the observed intervendor differences in FT strain measurements. cvi42 and Tomtec use optical flow methods, whereas Segment employs a rigid registration. Thereby, it is rather surprising that Segment and cvi42 had higher intramodality agreement they had versus Tomtec. Our study demonstrated that intervendor difference in FT algorithms is more important for regional than for global strains. In contrast to STE and tagging, the overall difficulty of FT analysis of regional strain is the absence of physical markers in the myocardium to follow and estimate regional deformation. Indeed, on regular cine bSSFP images, the myocardium is smooth, with no reliable difference in signal intensity that would aid in segmentation and tracking. As a result, FT software arbitrarily assign segments and tries tracking them based on probable movement, thus resulting in an overestimation of some segmental strains and underestimation of others, while correctly tracking the LV globally, where the detection of the blood-endocardial border is facilitated by the clear difference in intensity. It is, therefore, not surprising that regional strain analysis by FT is less performant than tagging. Nevertheless, regional strains were in closer agreement with tagging for CS than LS, probably because the circular nature of short-axis slices facilitates deformation estimates for FT. Another possibility could be that tagging is less accurate for LS evaluation, due to bad tag tracking at the base of the heart. This is suggested by the fact that agreement was, similar to our study comparing STE to tagging [12] least in inferior, infero-lateral and inferoseptal basal segments. Even though STE benefits from physical markers, in our present study, agreement of regional strains vs tagging and accuracy for detecting scar was better than that of STE. This is probably related to the fact that in this study, employing only one imaging modality with identical slices, the risk of misalignment of slices was less than in intermodality comparisons.

### Clinical implications

GLS, and, to a far lesser extent, GCS, have been investigated as potential prognostic biomarkers in various cardiac diseases, such as ischemic or non-ischemic cardiomyopathy [27–29], amyloidosis [30, 31], or hypertrophic cardiomyopathy [32]. Our study supports the overall accuracy of FT-CMR derived GLS and GCS with lower intervendor difference than previously reported for STE. Therefore, we believe for these 2 global strains, all software give sufficiently accurate results for clinical practice. By contrast, the important intervendor difference of GRS, requires further efforts in the standardization. Moreover, this implicates that normal values and cutoffs for strains are vendor dependent, and that follow-up studies should be conducted using the *same* analysis vendor. When using the same vendor overall interstudy reproducibility of FT global strains in other works was indeed excellent [20, 33, 34]. Overall, given the important variability in regional strains, we believe that the evidence accumulated so far is not solid enough to recommend using FT strain of any currently tested software for regional LV deformation analysis over other methods using physical markers of regional deformation such as tagging, DENSE or SENC.

## Limitations

Our study is limited by being a single-center study of relatively small size. The study was not balanced for gender and females were underrepresented particularly for ischemic heart disease. We did not evaluate torsion or layer-specific strain, as this option was not available for all software suites. Also, we did not evaluate right ventricular (RV) strain, as this is not possible with tagging due to the thin RV free wall. Likewise, we did not compare strain derivatives such as strain rate or time to peak. Because we only had access to 3 vendor software, not all commercial software was evaluated. Somewhat surprisingly the normal strains in our population were slightly lower than that in a large study reporting normal age and sex values of FT strain [35] and in a metanalysis of CMR derived normal FT strain [36], despite that the same software (Tomtec) was used in these studies as in our own. As compared to other works [7, 9–11], our study’s uniqueness but also limitation comes from the fact that we used 3T CMR. bSSFP cine images may more often be hampered by dark bands off-resonance artifacts at 3T than 1.5T, potentially affecting the accuracy of FT tracking and strain computation. Since we exerted a particular effort to good shimming, our overall image quality was good and we had few such artifacts and few tracking problems on the LV, as opposed to the RVy [34]. 3T also favors accuracy of tagging since tag persistence is better due to longer T1 times. An inherent limitation to all studies comparing segmental strains is that discrepancies can result in segmental misregistration among software. We, however, believe that this is very unlikely, as the same slice plane was imaged by tagging and cine bSSFP and the same anatomical markers have been used to define segments. Since tagging and bSSFP were performed in the same exam, changes in hemodynamic conditions are also unlikely to have affected results. We compared peak-systolic strain, as the exact temporal definition of end-systole in CMR is difficult. We did however not evaluate if peak-strain times were identical among software.

## Conclusion

In summary, our study demonstrated that 3 different FT software provides accurate values of GLS and GCS, with relatively minor differences among software or versus tagging. By contrast, significant intervendor differences in measurement and vs tagging were present for GRS. Also, on a regional basis, there was important variability of normal strain values for each vendor, and the difference in performance between software was substantial. Despite promising results for regional CS, variability was more important for regional LS and particularly for regional RS, suggesting that FT CMR may not be similarly reliable for regional deformation analysis, and that it requires further efforts for validation and standardization for regional FT strain analysis.

## Supplementary Information

**Additional file 1: Figure S1.** Bullseye graphs showing the absolute bias at regional level between FT and Tagging for LS (a) CS (b) and RS (c) in the study population.

**Additional file 2: Figure S2.** Receiver operating characteristics curve analysis comparing diagnostic abilities of detection of different degrees ≥ 25%, ≥ 50%, ≥ 75% LGE) of infarcted segments by regional LS, CS, and RS by tagging and the 3 FT software.

## Data Availability

The datasets used and/or analyzed during the current study are available from the corresponding author on reasonable request.

## Abbreviations

BP: Blood pressure
BSA: Body surface area
bSSFP: Balanced steady state free precession
CAD: Coronary artery disease
CI: Confidence interval
CMR: Cardiovascular magnetic resonance
CS: Circumferential strain
DBP: Diastolic blood pressure
DCM: Dilated cardiomyopathy
ECG: Electrocardiogram
FT: Feature tracking
GCS: Global circumferential strain
GFR: Glomerular filtration rate
GLS: Global longitudinal strain
GRS: Global radial strain
HF: Heart failure
HFrEF: Heart failure with reduced ejection fraction
ISCH: Ischemic cardiomyopathy group
HR: Hazard ratio
LGE: Late gadolinium enhancement
LS: Longitudinal strain
LV: Left ventricle/left ventricular
LVEDV: Left ventricular end-diastolic volume
LVH: Left ventricular hypertrophy
LVEF: Left ventricular ejection fraction
LVESV: Left ventricular end-systolic volume
RS: Radial strain
RV: Right ventricle/right ventricular
SBP: Systolic blood pressure
SPAMM: Spatial modulation of magnetization
STE: Speckle tracking echocardiography
VOL: Healthy group

## References

[1] Amzulescu MS, De Craene M, Langet H, Pasquet A, Vancraeynest D, Pouleur AC (2019). Myocardial strain imaging: review of general principles, validation, and sources of discrepancies. Eur Heart J Cardiovasc Imaging..

[2] Claus P, Omar AMS, Pedrizzetti G, Sengupta PP, Nagel E (2015). Tissue tracking technology for assessing cardiac mechanics: principles, normal values, and clinical applications. JACC Cardiovasc Imaging..

[3] Farsalinos KE, Daraban AM, Unlu S, Thomas JD, Badano LP, Voigt JU (2015). Head-to-head comparison of global longitudinal strain measurements among nine different vendors: the EACVI/ASE Inter-Vendor Comparison Study. J Am Soc Echocardiography.

[4] Nagata Y, Takeuchi M, Mizukoshi K, Wu VC, Lin FC, Negishi K (2015). Intervendor variability of two-dimensional strain using vendor-specific and vendor-independent software. J Am Soc Echocardiography.

[5] Risum N, Ali S, Olsen NT, Jons C, Khouri MG, Lauridsen TK (2012). Variability of global left ventricular deformation analysis using vendor dependent and independent two-dimensional speckle-tracking software in adults. J Am Soc Echocardiography.

[6] Voigt JU, Pedrizzetti G, Lysyansky P, Marwick TH, Houle H, Baumann R (2015). Definitions for a common standard for 2D speckle tracking echocardiography: consensus document of the EACVI/ASE/Industry Task Force to standardize deformation imaging. J Am Soc Echocardiography.

[7] Cao JJ, Ngai N, Duncanson L, Cheng J, Gliganic K, Chen Q (2018). A comparison of both DENSE and feature tracking techniques with tagging for the cardiovascular magnetic resonance assessment of myocardial strain. J Cardiovasc Magnetic Resonance..

[8] Bucius P, Erley J, Tanacli R, Zieschang V, Giusca S, Korosoglou G, et al. Comparison of feature tracking, fast-SENC, and myocardial tagging for global and segmental left ventricular strain. ESC Heart Failure. 2019. (Epub 2019/12/05. eng). 10.1002/ehf2.12576 PMC7160507 31800152

[9] Gertz RJ, Lange T, Kowallick JT, Backhaus SJ, Steinmetz M, Staab W (2018). Inter-vendor reproducibility of left and right ventricular cardiovascular magnetic resonance myocardial feature-tracking. PloS one..

[10] Barreiro-Pérez M, Curione D, Symons R, Claus P, Voigt JU, Bogaert J (2018). Left ventricular global myocardial strain assessment comparing the reproducibility of four commercially available CMR-feature tracking algorithms. Eur Radiol..

[11] Dobrovie M, Barreiro-Pérez M, Curione D, Symons R, Claus P, Voigt JU (2019). Inter-vendor reproducibility and accuracy of segmental left ventricular strain measurements using CMR feature tracking. Eur Radiol..

[12] Amzulescu MS, Langet H, Saloux E, Manrique A, Boileau L, Slimani A, et al. Head-to-head comparison of global and regional two-dimensional speckle tracking strain versus cardiac magnetic resonance tagging in a multicenter validation study. Circulation Cardiovasc Imaging. 2017;10(11):e006530. 10.1161/CIRCIMAGING.117.006530 (**Epub 2017/11/16. eng**). 10.1161/CIRCIMAGING.117.006530 29138230

[13] Amzulescu MS, Rousseau MF, Ahn SA, Boileau L, de Ravenstein MC, Vancraeynest D (2015). Prognostic impact of hypertrabeculation and noncompaction phenotype in dilated cardiomyopathy: a CMR study. JACC Cardiovasc Imaging..

[14] Moody WE, Taylor RJ, Edwards NC, Chue CD, Umar F, Taylor TJ (2015). Comparison of magnetic resonance feature tracking for systolic and diastolic strain and strain rate calculation with spatial modulation of magnetization imaging analysis. J Magnetic Resonance Imaging.

[15] Ohyama Y, Ambale-Venkatesh B, Chamera E, Shehata ML, Corona-Villalobos CP, Zimmerman SL (2015). Comparison of strain measurement from multimodality tissue tracking with strain-encoding MRI and harmonic phase MRI in pulmonary hypertension. Int J Cardiol.

[16] Lu JC, Connelly JA, Zhao L, Agarwal PP, Dorfman AL (2014). Strain measurement by cardiovascular magnetic resonance in pediatric cancer survivors: validation of feature tracking against harmonic phase imaging. Pediat Radiol.

[17] Kuetting DLR, Feisst A, Dabir D, Homsi R, Sprinkart AM, Luetkens J (2017). Comparison of magnetic resonance feature tracking with CSPAMM HARP for the assessment of global and regional layer specific strain. Int J Cardiol.

[18] Hor KN, Gottliebson WM, Carson C, Wash E, Cnota J, Fleck R (2010). Comparison of magnetic resonance feature tracking for strain calculation with harmonic phase imaging analysis. JACC Cardiovasc Imaging..

[19] Augustine D, Lewandowski AJ, Lazdam M, Rai A, Francis J, Myerson S (2013). Global and regional left ventricular myocardial deformation measures by magnetic resonance feature tracking in healthy volunteers: comparison with tagging and relevance of gender. J Cardiovasc Magnetic Reson.

[20] Singh A, Steadman CD, Khan JN, Horsfield MA, Bekele S, Nazir SA (2015). Intertechnique agreement and interstudy reproducibility of strain and diastolic strain rate at 15 and 3 Tesla: a comparison of feature-tracking and tagging in patients with aortic stenosis. J Magnetic Reson Imaging..

[21] Graham-Brown MP, Gulsin GS, Parke K, Wormleighton J, Lai FY, Athithan L (2019). A comparison of the reproducibility of two cine-derived strain software programmes in disease states. Eur J Radiol.

[22] Wehner GJ, Jing L, Haggerty CM, Suever JD, Chen J, Hamlet SM (2018). Comparison of left ventricular strains and torsion derived from feature tracking and DENSE CMR. J Cardiovas Magnetic Reson.

[23] Amzulescu M, Langet H, Saloux E, Manrique A, Slimani A, Allain P, et al. Validation of global and regional 3D speckle tracking strain vs 3D cardiac magnetic resonance tagging. Eur Heart J. 2017 2017. 10.1161/CIRCIMAGING.117.006530 29138230

[24] Massie BM, Fisher SG, Radford M, Deedwania PC, Singh BN, Fletcher RD (1996). Effect of amiodarone on clinical status and left ventricular function in patients with congestive heart failure CHF-STAT investigators. Circulation.

[25] Harrild DM, Han Y, Geva T, Zhou J, Marcus E, Powell AJ (2012). Comparison of cardiac MRI tissue tracking and myocardial tagging for assessment of regional ventricular strain. Int J Cardiovasc Imaging..

[26] Wu L, Germans T, Guclu A, Heymans MW, Allaart CP, van Rossum AC (2014). Feature tracking compared with tissue tagging measurements of segmental strain by cardiovascular magnetic resonance. J Cardiovasc Magnetic Reson.

[27] Romano S, Judd RM, Kim RJ, Kim HW, Klem I, Heitner JF (2018). Feature-tracking global longitudinal strain predicts death in a multicenter population of patients with ischemic and nonischemic dilated cardiomyopathy incremental to ejection fraction and late gadolinium enhancement. JACC Cardiovasc Imaging..

[28] Mordi I, Bezerra H, Carrick D, Tzemos N (2015). The combined incremental prognostic value of LVEF, late gadolinium enhancement, and global circumferential strain assessed by CMR. JACC Cardiovasc Imaging..

[29] Riffel JH, Keller MG, Rost F, Arenja N, Andre F, Siepen AF (2016). Left ventricular long axis strain: a new prognosticator in non-ischemic dilated cardiomyopathy?. J Cardiovasc Magnetic Reson.

[30] Bhatti S, Vallurupalli S, Ambach S, Magier A, Watts E, Truong V (2018). Myocardial strain pattern in patients with cardiac amyloidosis secondary to multiple myeloma: a cardiac MRI feature tracking study. Int J Cardiovasc Imaging..

[31] Kuetting DL, Homsi R, Sprinkart AM, Luetkens J, Thomas DK, Schild HH (2017). Quantitative assessment of systolic and diastolic function in patients with LGE negative systemic amyloidosis using CMR. Int J Cardiol.

[32] Saito M, Okayama H, Yoshii T, Higashi H, Morioka H, Hiasa G (2012). Clinical significance of global two-dimensional strain as a surrogate parameter of myocardial fibrosis and cardiac events in patients with hypertrophic cardiomyopathy. Eur Heart J Cardiovasc Imaging..

[33] Morton G, Schuster A, Jogiya R, Kutty S, Beerbaum P, Nagel E (2012). Inter-study reproducibility of cardiovascular magnetic resonance myocardial feature tracking. J Cardiovasc Magnetic Reson.

[34] Houard L, Militaru S, Tanaka K, Pasquet A, Vancraeynest D, Vanoverschelde JL, et al. Test-retest reliability of left and right ventricular systolic function by new and conventional echocardiographic and cardiac magnetic resonance parameters. Eur Heart J Cardiovasc Imaging. 2020(in press). Epub 2020/08/15. 10.1093/ehjci/jeaa206 32793957

[35] Taylor RJ, Moody WE, Umar F, Edwards NC, Taylor TJ, Stegemann B (2015). Myocardial strain measurement with feature-tracking cardiovascular magnetic resonance: normal values. Eur Heart J Cardiovasc Imaging..

[36] Vo HQ, Marwick TH, Negishi K (2018). MRI-derived myocardial strain measures in normal subjects. JACC Cardiovasc Imaging..

