# Supplementary material for: Multivendor comparison of global and regional 2D cardiovascular magnetic resonance feature tracking strains vs tissue tagging at 3T
Source: J Cardiovasc Magn Reson. 2021 May 13;23:54. doi: 10.1186/s12968-021-00742-3 (PMC8117295; doi:10.1186/s12968-021-00742-3)

Bias Regional longitudinal strains

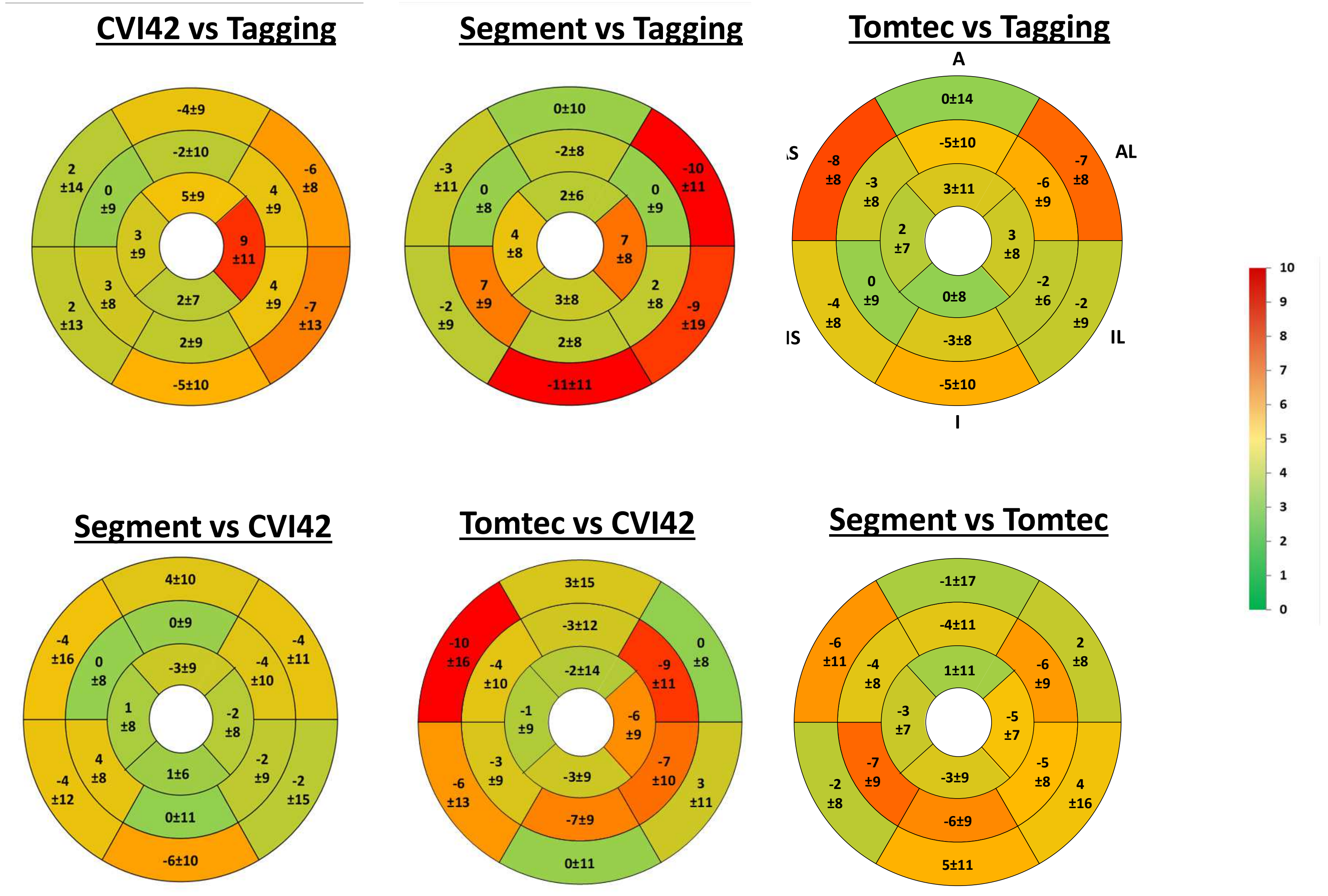

Bias Regional radial strains

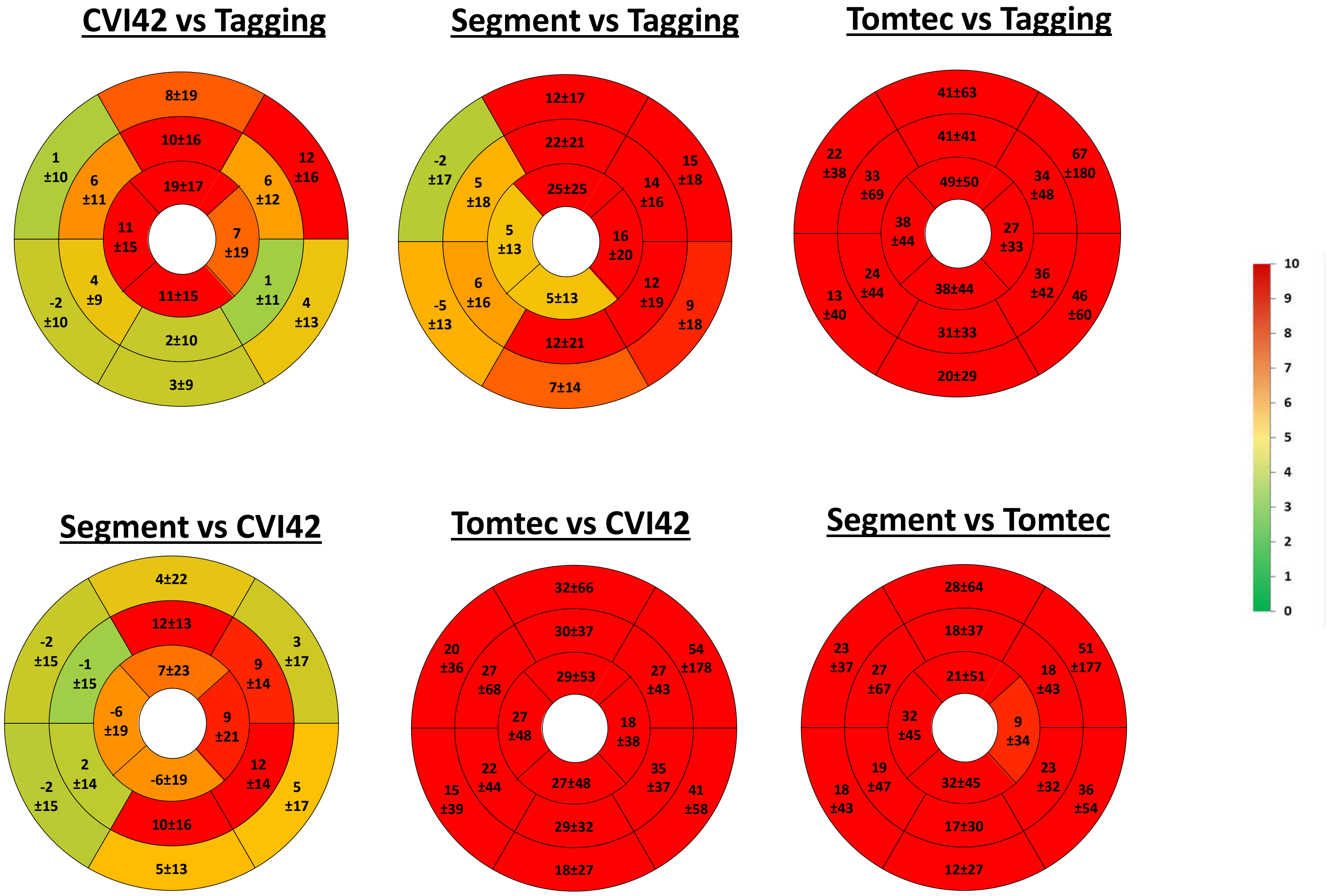

Bias Regional circumferential strains

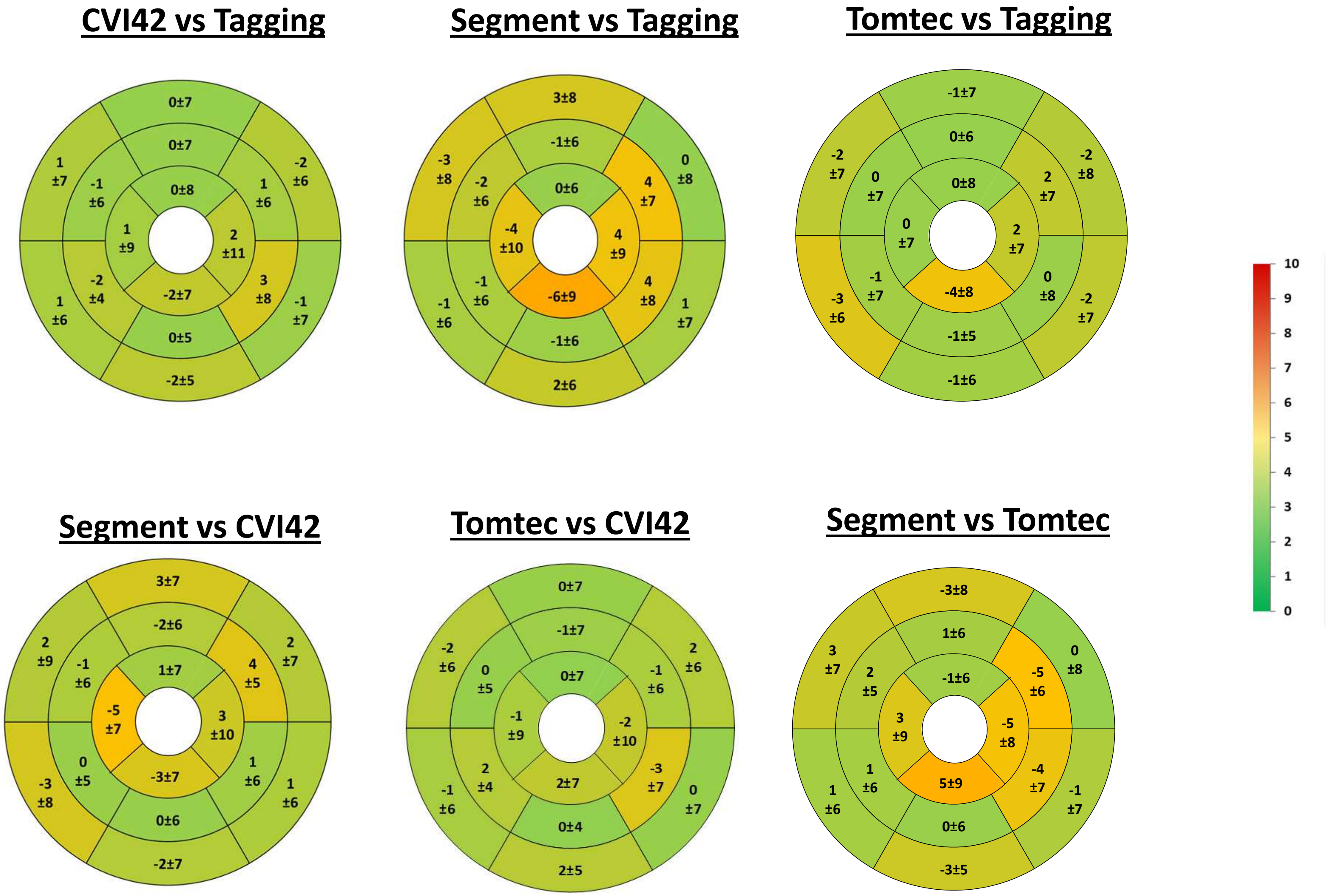

Supplement: Supplementary file 1 — Additional file 1: Figure S1. Bullseye graphs showing the absolute bias at regional level between FT and Tagging for LS (a) CS (b) and RS (c) in the study population. [file 12968_2021_742_MOESM1_ESM.pdf]
