# Supplementary figures and images for: Multivendor comparison of global and regional 2D cardiovascular magnetic resonance feature tracking strains vs tissue tagging at 3T
Source: J Cardiovasc Magn Reson. 2021 May 13;23:54. doi: 10.1186/s12968-021-00742-3 (PMC8117295; doi:10.1186/s12968-021-00742-3)

## LGE >25% transmural

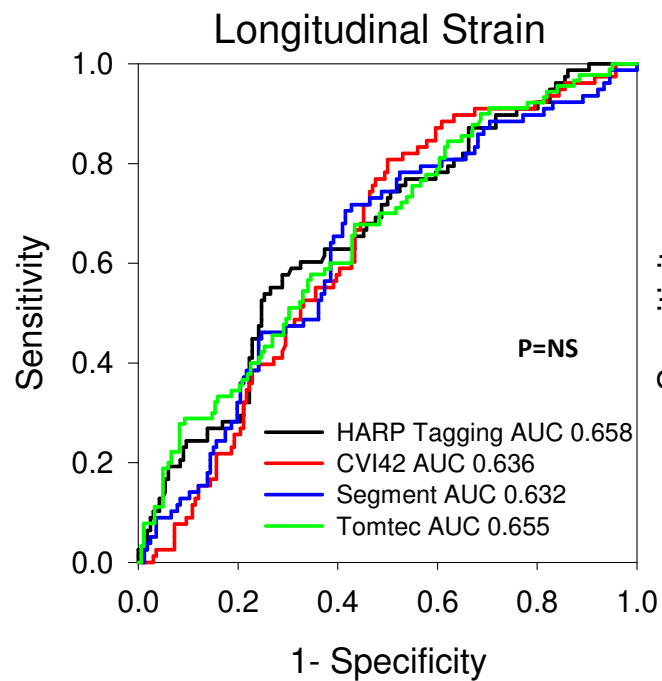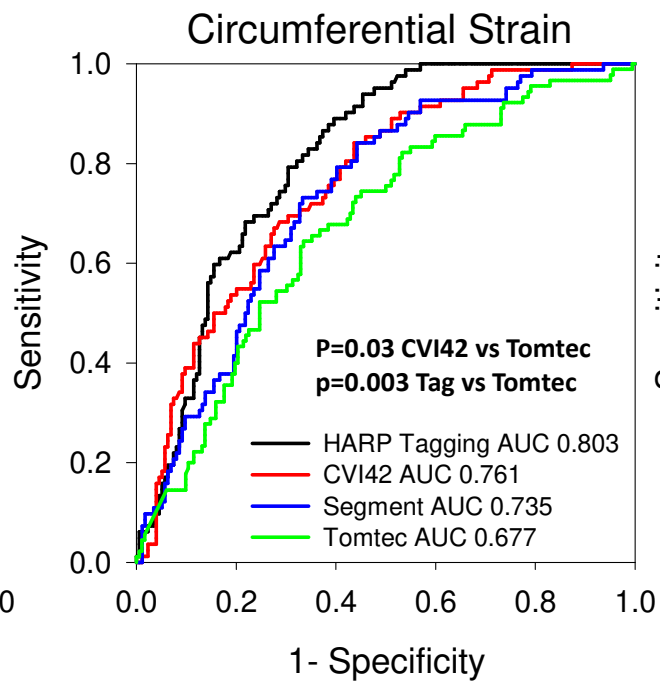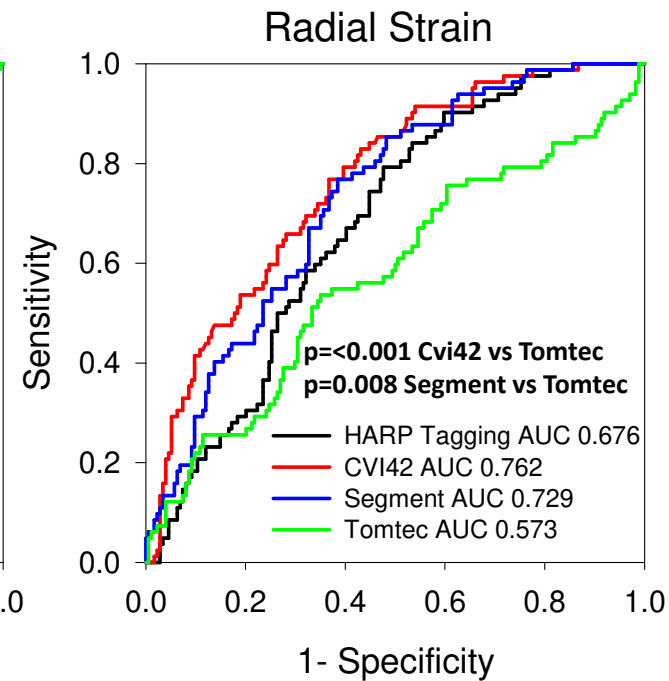

## LGE >75% transmural

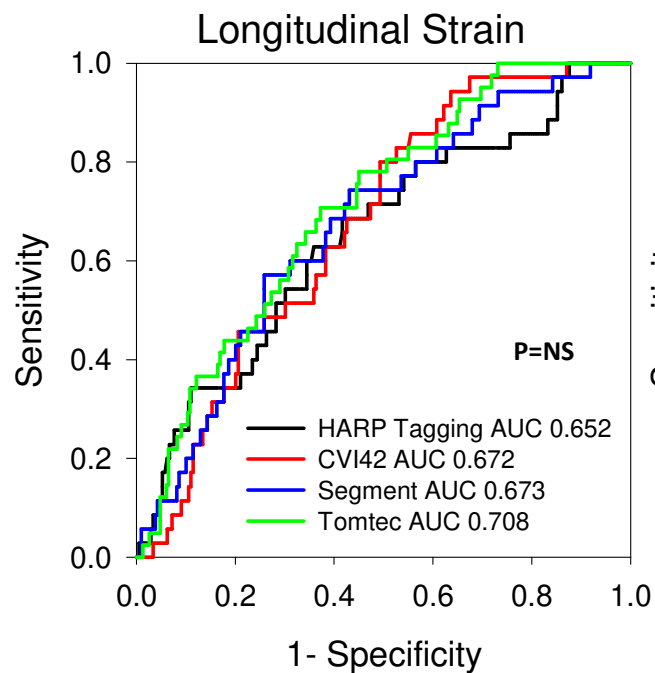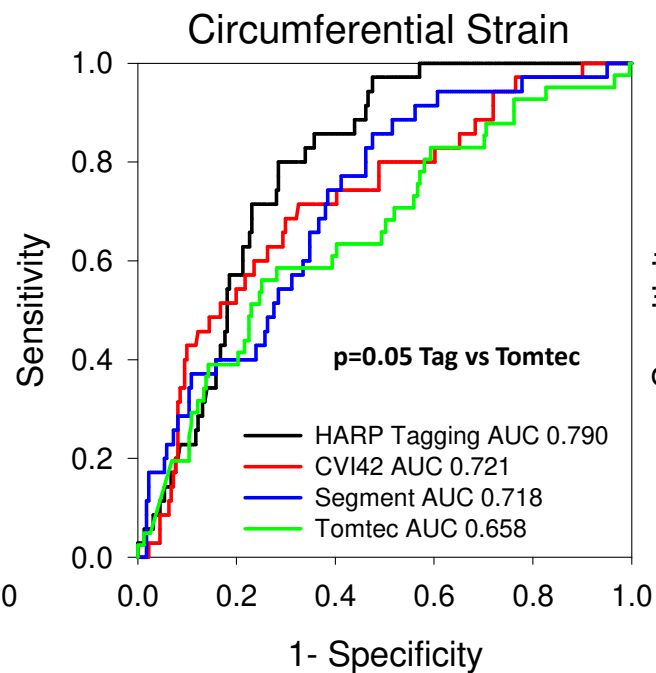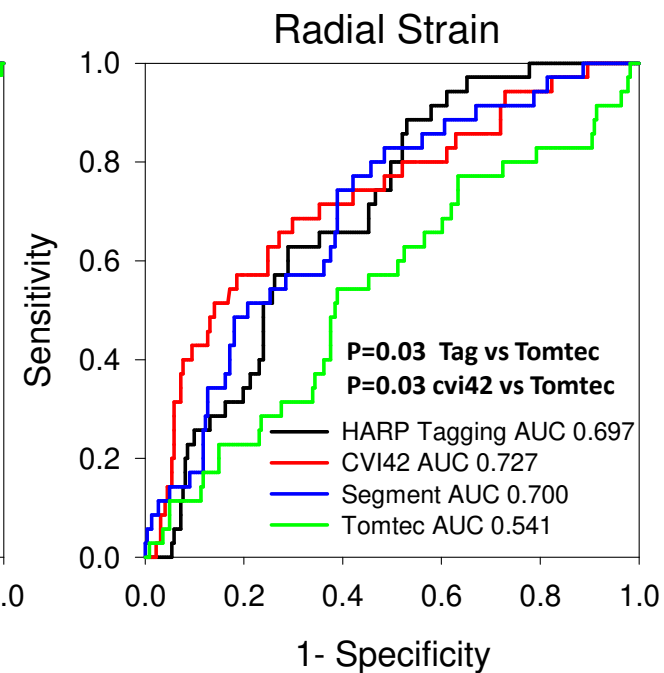

## LGE >50% transmural

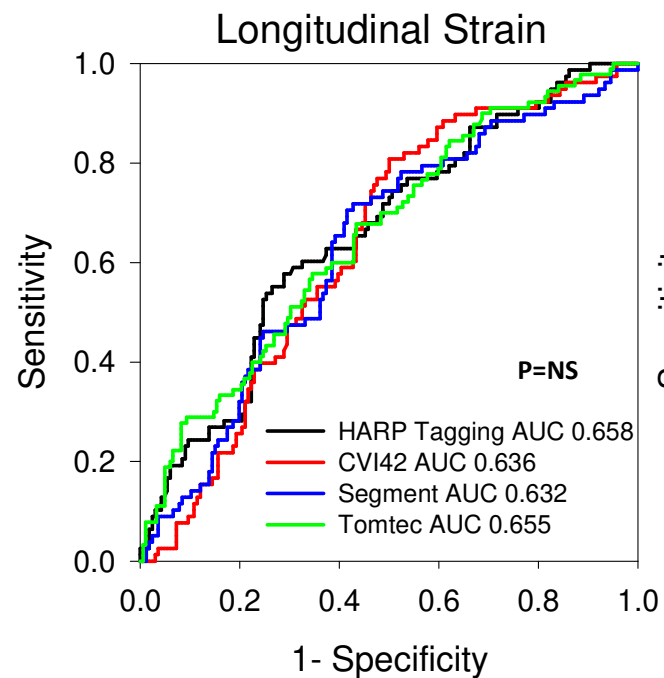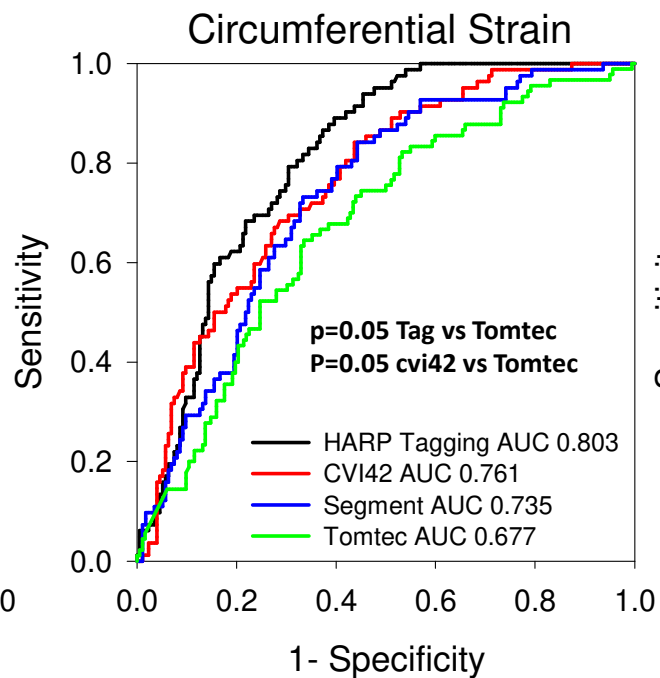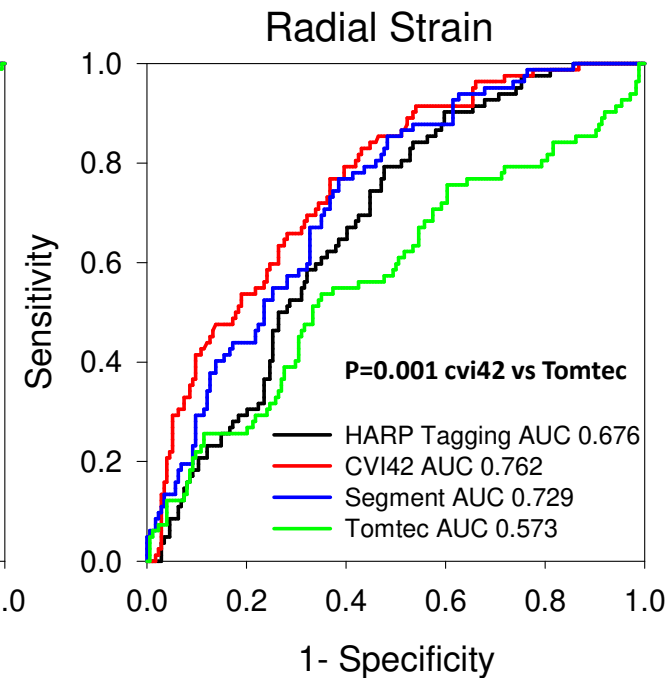

Supplement: Supplementary file 2 — Additional file 2: Figure S2. Receiver operating characteristics curve analysis comparing diagnostic abilities of detection of different degrees ≥ 25%, ≥ 50%, ≥ 75% LGE) of infarcted segments by regional LS, CS, and RS by tagging and the 3 FT software. [file 12968_2021_742_MOESM2_ESM.pdf]
